# A Rare Case of Post-orchidectomy Arterial Injury With Rapidly Enlarging Scrotal Hematoma Treated With Coil Embolization

**DOI:** 10.7759/cureus.47914

**Published:** 2023-10-29

**Authors:** Diljot Dhillon, Harrison V Moynihan, Giovanni Santoro, Kenny Lien, Michael J Dayan

**Affiliations:** 1 Interventional Radiology, Zucker School of Medicine at Hofstra/Northwell at Mather Hospital, Port Jefferson, USA

**Keywords:** hematoma, scrotal, arterial, embolization, orchidectomy

## Abstract

Testicular cancer is the most common solid tumor in young adult males. Radical inguinal orchidectomy is the gold standard for the diagnosis and treatment of testicular cancer, which is confined to the scrotum and is generally well tolerated. An uncommon, but known, complication of radical orchidectomy is scrotal hematoma. Scrotal hematoma from radical orchidectomy is commonly self-limited and typically self-resolving.

We present a rare case of metastatic testicular malignancy diagnosed with radical inguinal orchidectomy complicated by a rapidly enlarging scrotal hematoma, successfully treated with surgical evacuation and image-guided arterial embolization.

## Introduction

Testicular cancer represents only 1% of all tumors in males and 5% of all urological malignancies [1]. However, it is the most common solid malignancy in young adult men 15 to 40 years of age [2,3]. With current advancements in care, it is one of the most treatable malignancies with a 97% five-year survival rate [4]. There are multiple different histopathologic subtypes, with germ cell tumors accounting for 95% of all cases, of which the majority are derived from germ cell neoplasia in situ (GCNIS) [1]. Risk factors include cryptorchidism and family history of testicular malignancy [4].

Initial imaging evaluation is done with scrotal ultrasound, which has demonstrated a 92-98% sensitivity and 95-99% specificity for identifying testicular malignancy [5]. Biochemical evaluation with serum tumor markers alpha-fetoprotein (AFP), lactate dehydrogenase (LDH), and human chorionic gonadotropin (hCG) is performed to aid in diagnosis. Radical inguinal orchidectomy is the gold standard for diagnosis and treatment of local testicular malignancy [1]. The surgical approach involves inguinal excision, extraction of the testicle, and ligation of the spermatic cord at the inguinal ring [1].

Scrotal hematomas are a known complication of radical orchidectomy, with an incidence of 1-2% of all cases. These hematomas are commonly self-resolving and do not require intervention [1]. We present a rare case of a post-orchidectomy arterial injury resulting in a rapidly enlarging scrotal hematoma. Our case presents a rare source of bleeding into the scrotum from collateral vessels supplied by the median circumflex femoral artery and profunda femoral artery. The authors could not find other examples of this phenomenon documented in current literature. Furthermore, we discuss the role of cross-sectional imaging, conventional angiography, and embolization in identifying and treating active bleeding sources that are not identified during surgical hematoma evacuation.

## Case presentation

A 21-year-old male with a past medical history of congenital aortic stenosis presented to the emergency department with complaints of a two-month history of lower back pain, and painless right testicular swelling.

**Figure 1:**
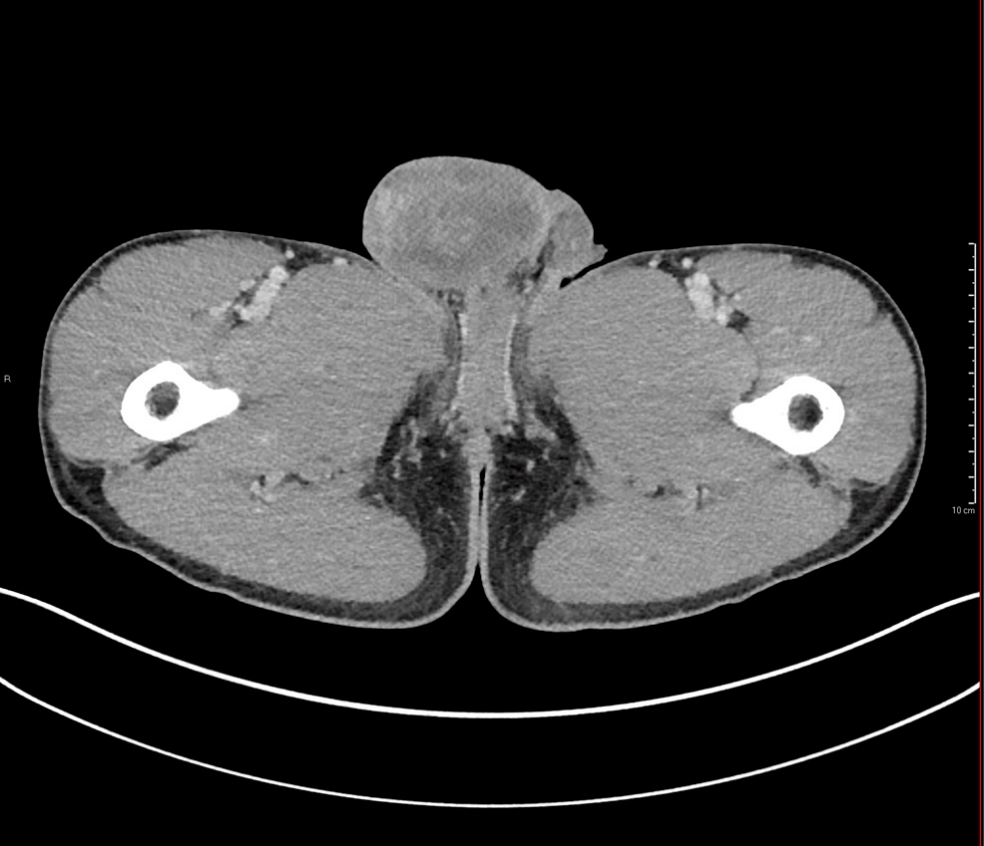
Axial intravenous contrast-enhanced computed tomography image of the abdomen and pelvis demonstrating a heterogeneous right testicular mass

A physical exam revealed bilateral inguinal lymphadenopathy and a firm non-tender right testicular mass. On admission, an abdomen and pelvis computed tomography was performed that revealed a large right testicular mass (Figure 1).

**Figure 2:**
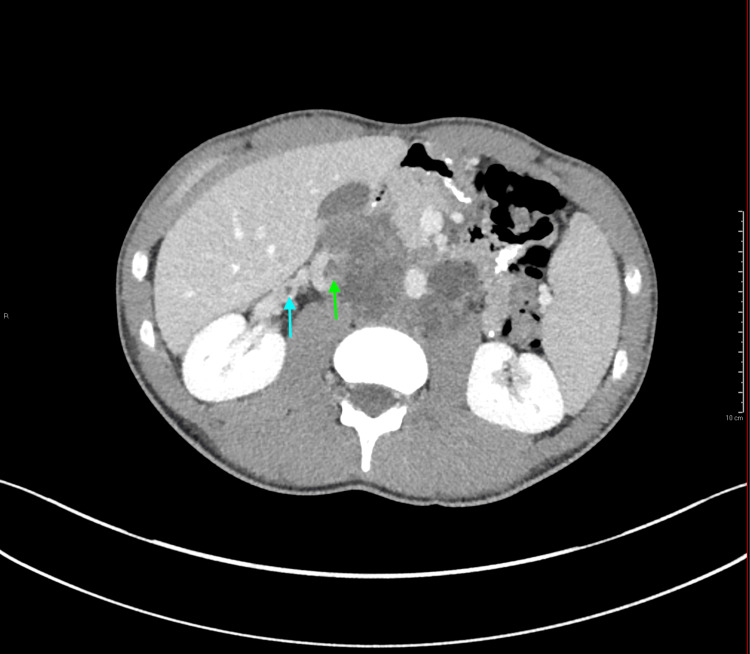
Axial intravenous contrast-enhanced computed tomography image of the abdomen and pelvis demonstrating retroperitoneal lymphadenopathy, with tumor extension into the intrahepatic inferior vena cava (green arrow) and right gonadal vein (blue arrow) These findings are concerning for metastatic testicular malignancy.

Also identified on CT was retroperitoneal lymphadenopathy, as well as a tumor thrombus within the intrahepatic inferior vena cava (IVC) with extension into the right gonadal vein (Figure 2). Bilateral lung metastases were also identified (not pictured). No abnormally enlarged inguinal lymph nodes were seen on the CT scan.

**Figure 3:**
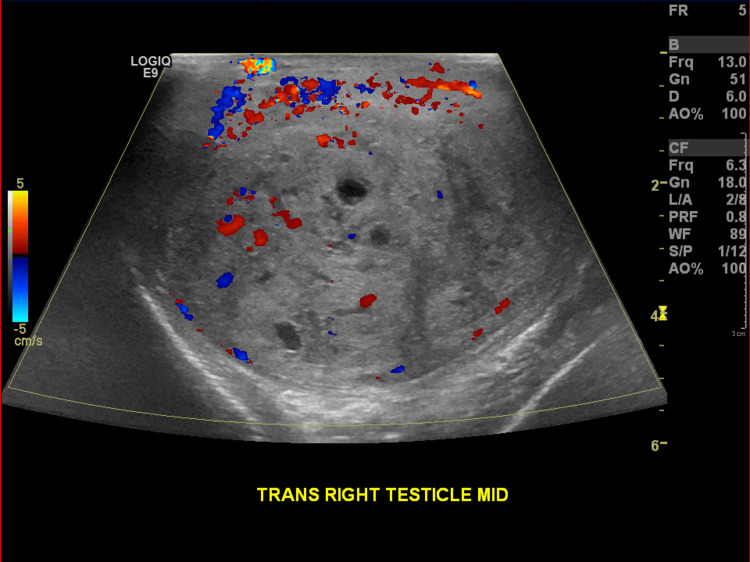
Sonographic image of the scrotum demonstrating a large heterogenous mass replacing the right testicle The mass demonstrates internal vascularity, calcifications, and cystic spaces.

Subsequently, a testicular ultrasound was obtained that revealed an 8.2 cm heterogenous mass replacing the right testicle with internal vascularity, coarse calcifications, and cystic spaces (Figure 3).

On Day 2 of the hospital course, the patient underwent a right inguinal approach radical orchidectomy. The surgery was performed by an experienced urologist, with documented secured hemostasis, minimal reported blood loss, and no complications. He was subsequently discharged on postoperative day (POD) 1/Day 3 of his hospital course. He returned to the emergency department within 24 hours due to increased scrotal pain and right-sided scrotal swelling.

**Figure 4:**
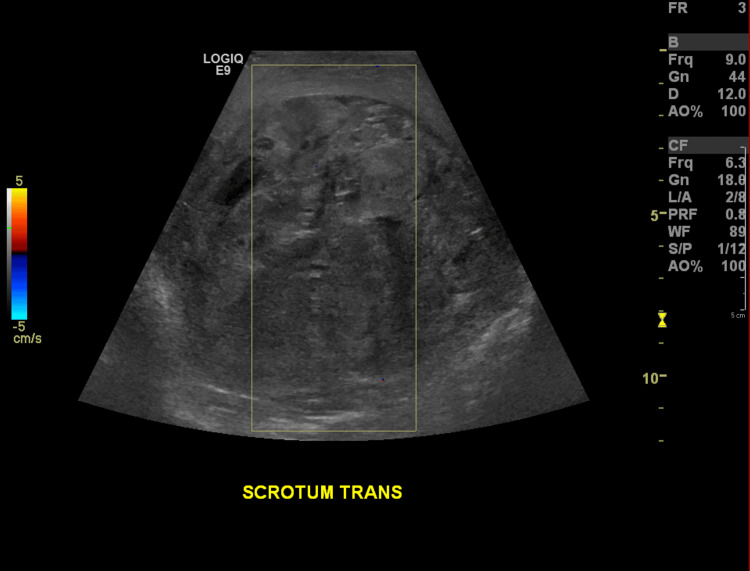
Sonographic image of the right testicle status post-orchidectomy demonstrating a large heterogenous collection within the surgical bed

Upon re-presentation to the ED, a testicular ultrasound was performed that revealed a heterogeneous collection within the surgical bed, consistent with a hematoma, measuring 11.4 x 8.3 cm (Figure 4).

**Figure 5:**
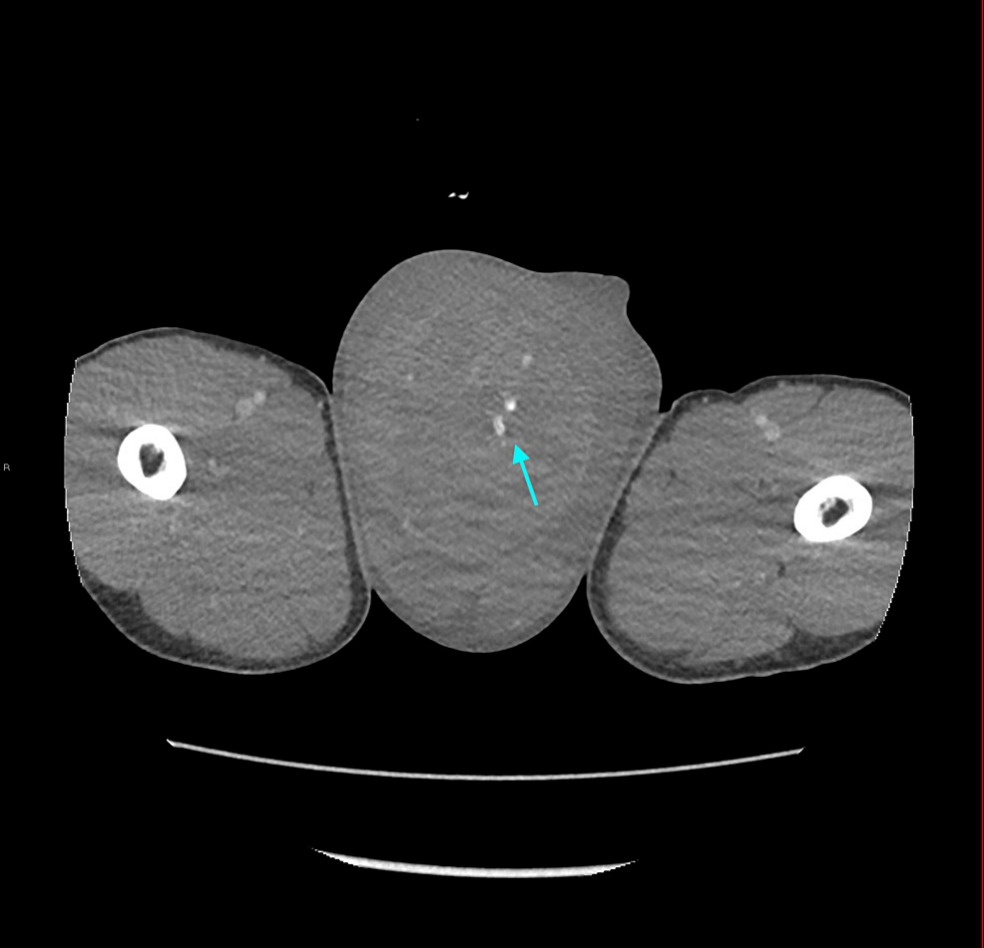
Contrast-enhanced CTA of the abdomen and pelvis demonstrating a large heterogenous collection within the right scrotum consistent with a hematoma, with evidence of active contrast extravasation (arrow) CTA: computed tomography angiography

**Figure 6:**
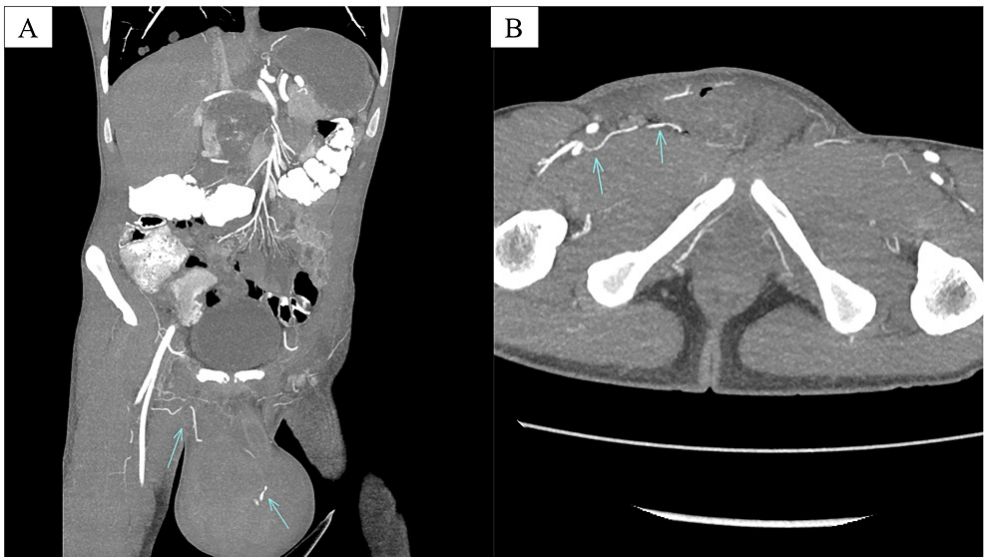
Coronal (A) and axial (B) maximal intensity projection images in the arterial phase from the contrast-enhanced CTA of the abdomen and pelvis demonstrating a large heterogenous collection within the right scrotum consistent with a hematoma, with evidence of active contrast extravasation. The arterial supply can be traced to the profunda femoris artery (arrows). CTA: computed tomography angiography

A computed tomography angiography (CTA) of the abdomen and pelvis was subsequently performed revealing an 11.4 cm heterogeneous collection within the right scrotum consistent with a hematoma with evidence of active extravasation seen on arterial phase imaging (Figure 5). The arterial supply was traced to a branch of the profunda femoris artery (Figure 6).

The patient immediately underwent a surgical hematoma evacuation without a definite bleeding source identified during surgery. The patient was thereafter transferred to interventional radiology for identification and embolization of the bleeding source.

**Figure 7:**
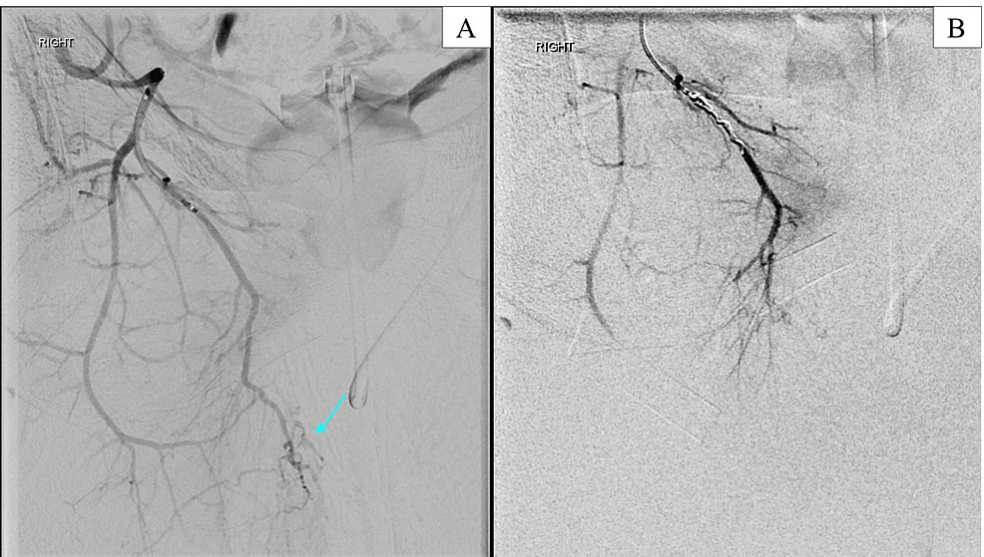
(A) Sub-selective digital subtraction angiography (DSA) of the medial circumflex femoral artery demonstrating collateral supply to the scrotum (blue arrow). (B): Post-embolization DSA images of the medial circumflex femoral artery that no longer demonstrates collateral vessels to the scrotum.

A right external iliac angiogram was performed that revealed a variant origin of the medial circumflex femoral artery, arising from the common femoral artery. Sub-selective angiography of the variant medial circumflex femoral artery revealed collateral supply to the scrotum with evidence of active arterial extravasation. A microcatheter was advanced over the wire into the branch of the medial circumflex femoral artery supplying collateral circulation to the scrotum, and the branch was embolized using detachable micro-coils (Figure 7).

**Figure 8:**
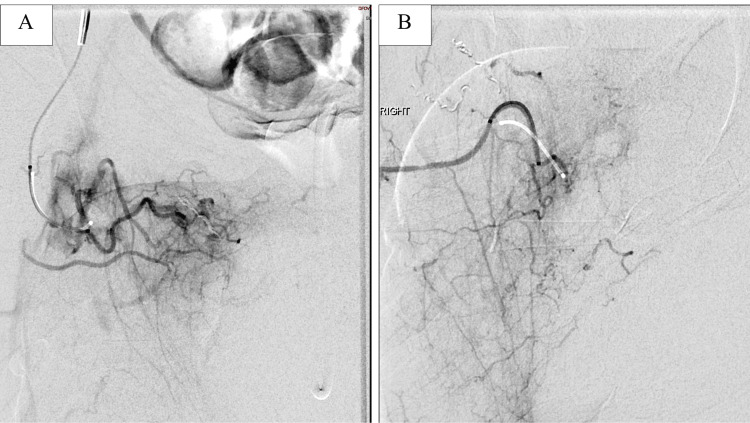
Sub-selective digital subtraction angiography (DSA) images of the second medial perforator branch (A) and third medial perforator branch (B) of the right profunda femoral artery with active arterial extravasation into the scrotum.

After embolization of the medial circumflex branches, sub-selective angiography was performed of the profunda femoral artery and its perforating branches that demonstrated active arterial extravasation into the scrotum from the second and third medial perforator branches (Figure 8).

**Figure 9:**
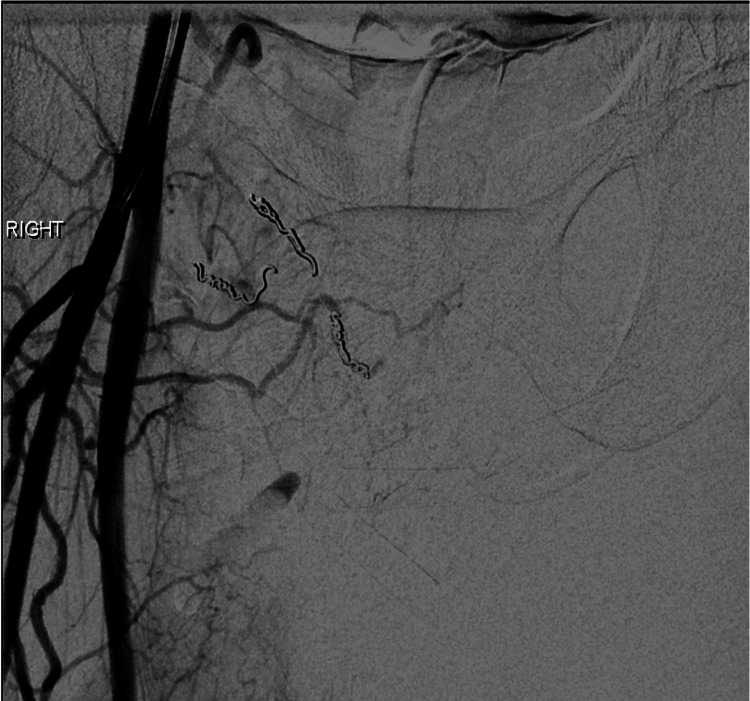
Final digital subtraction angiography (DSA) image of the common femoral artery with no evidence of arterial extravasation from the medial circumflex artery or the perforator branches of the profunda femoral artery into the scrotum

Successful coil embolization of the second and third medial perforator branches of the right profunda femoral artery was performed also using detachable microcoils. Post-embolization angiography demonstrated no active extravasation (Figure 9). 

After treatment with surgical evacuation and arterial embolization, the patient’s scrotal swelling resolved. There were no complications from angioembolization. The patient remained admitted for three hospital days for postoperative monitoring. The scrotal hematoma did not reaccumulate. The patient was thereafter transferred to a tertiary care center for a higher level of oncologic care.

Surgical pathology revealed a malignant testicular non-seminomatous mixed germ cell tumor, with mixed components of yolk sac tumor and embryonal sarcoma. Immunohistochemical studies were positive for SALL4, OCT4, pancytokeratin, and alpha-fetoprotein.

The greatest dimension of the main tumor mass was measured at 8.0 x 6.0 x 4.5 cm, extending beyond the tunica albuginea and involving the adjacent soft tissue. Multifocal lymphovascular invasion was also present, with the involvement of three lymph nodes within the soft tissues of the spermatic cord near the proximal resection margin. Pathologic stage classification per the American Joint Committee on Cancer (AJCC) 8th Edition Cancer Staging Manual was pT2 pN1 M1a.

## Discussion

The current gold standard for both the diagnosis and treatment of testicular cancer is radical inguinal orchidectomy [1]. Scrotal hematoma is a known complication of radical inguinal orchidectomy. Scrotal hematomas resulting from orchidectomy are commonly self-limited and self-resolving [1]. We present a rare case of scrotal hematoma, with arterial injury to a variant origin medial circumflex artery and to the second and third medial perforator branches of the profunda femoral artery. 

The normal testis receives arterial blood supply from the testicular artery that originates directly from the abdominal aorta at approximately the second to third lumbar vertebral level, just inferior to the origin of the renal arteries [6]. The testicular artery descends in the abdomen, passing through the deep inguinal ring, into the spermatic cord. The testicular artery will terminate, generally within the mediastinum testis, into the upper pole and lower pole segmental arteries. The upper pole segmental artery branches anastomose with branches of the artery of the vas deferens, whereas the lower pole segmental artery branches anastomose with terminal branches of the cremasteric artery [6]. Nearly all cases of testicular cancer are germ cell tumors that arise from the germ cell layer of the testes and receive blood supply from the testicular artery [7]. However, neovascularization has been described in clinical stage A testicular germ cell tumors, establishing the potential of extragonadal tumor angiogenesis [8].

In our case, the computed tomography angiography of the abdomen and pelvis identified arterial supply to the scrotal hematoma from perforating branches of the profunda femoris, with evidence of active arterial extravasation. Given the computed tomography angiography findings, during the conventional angiography, the right external iliac artery, common femoral artery, and profunda femoris were all evaluated. The right external artery angiogram demonstrated a variant origin of the medial circumflex artery arising from the common femoral artery, as opposed to its normal origin from the profunda femoris [9]. The medial circumflex artery branches normally supply the femoral neck, adductor muscles, and hamstring muscles, but do not have branches supplying the scrotum. Our patient had extensive collateral vessels originating from the medial circumflex femoral artery, supplying the right hemiscrotum, with evidence of active extravasation. Multiple collateral vessels from the second and third deep perforator branches of the profunda femoris were also identified and found to have active arterial extravasation into the right hemiscrotum. The arterial supply of the testes from either the medial circumflex artery or the profunda femoris is an uncommon phenomenon. We surmise that the testicular malignancy, given its large size and distant metastases, may have received arterial supply from these collateral vessels via neovascularization. Upon removal of the testis, damage to these fragile neovessels likely resulted in arterial bleeding and rapid formation of a scrotal hematoma.

## Conclusions

When performing radical orchidectomy, along with carefully ligating the spermatic cord, it is critical to meticulously identify additional arterial sources of bleeding to prevent the formation of a scrotal hematoma. If a patient presents with a rapidly progressive enlarging scrotum after radical inguinal orchidectomy, it is imperative to consider arterial injury as the source of the scrotal hematoma. Arterial injury is not limited to the testicular artery and its branches and can also involve arteries in the pelvis and/or lower extremity. Cross-sectional arterial phase imaging of the abdomen and pelvis can confirm the presence of arterial extravasation into the scrotum and aid in localizing the source of the bleeding. Angiography can be used for definitive identification of injured vessels that can be subsequently treated with embolization.
